# *EVI1* Inhibits Apoptosis Induced by Antileukemic Drugs via Upregulation of *CDKN1A/p21/WAF* in Human Myeloid Cells

**DOI:** 10.1371/journal.pone.0056308

**Published:** 2013-02-14

**Authors:** Anna Rommer, Birgit Steinmetz, Friederike Herbst, Hubert Hackl, Petra Heffeter, Daniela Heilos, Martin Filipits, Katarina Steinleitner, Shayda Hemmati, Irene Herbacek, Ilse Schwarzinger, Katharina Hartl, Pieter Rondou, Hanno Glimm, Kadin Karakaya, Alwin Krämer, Walter Berger, Rotraud Wieser

**Affiliations:** 1 Department of Medicine I, Medical University of Vienna, Vienna, Austria; 2 Comprehensive Cancer Center of the Medical University of Vienna, Vienna, Austria; 3 Department of Translational Oncology, National Center for Tumor Diseases and German Cancer Research Center, Heidelberg, Germany; 4 Division of Bioinformatics, Innsbruck Medical University, Innsbruck, Austria; 5 Department of Medicine I, Institute of Cancer Research, and Research Platform “Translational Cancer Therapy Research”, Medical University of Vienna, Vienna, Austria; 6 Department of Laboratory Medicine, Medical University of Vienna, Vienna, Austria; 7 Center for Medical Genetics Ghent, Ghent University Hospital Medical Research Building, Ghent, Belgium; 8 Clinical Cooperation Unit Molecular Haematology/Oncology, German Cancer Research Center, Heidelberg, Germany; Institut national de la santé et de la recherche médicale (INSERM), France

## Abstract

Overexpression of *ecotropic viral integration site 1* (*EVI1*) is associated with aggressive disease in acute myeloid leukemia (AML). Despite of its clinical importance, little is known about the mechanism through which *EVI1* confers resistance to antileukemic drugs. Here, we show that a human myeloid cell line constitutively overexpressing *EVI1* after infection with a retroviral vector (U937_EVI1) was partially resistant to etoposide and daunorubicin as compared to empty vector infected control cells (U937_vec). Similarly, inducible expression of *EVI1* in HL-60 cells decreased their sensitivity to daunorubicin. Gene expression microarray analyses of U937_EVI1 and U937_vec cells cultured in the absence or presence of etoposide showed that 77 and 419 genes were regulated by *EVI1* and etoposide, respectively. Notably, mRNA levels of 26 of these genes were altered by both stimuli, indicating that *EVI1* regulated genes were strongly enriched among etoposide regulated genes and vice versa. One of the genes that were induced by both *EVI1* and etoposide was *CDKN1A/p21/WAF*, which in addition to its function as a cell cycle regulator plays an important role in conferring chemotherapy resistance in various tumor types. Indeed, overexpression of *CDKN1A* in U937 cells mimicked the phenotype of *EVI1* overexpression, similarly conferring partial resistance to antileukemic drugs.

## Introduction

Aberrant expression of the *ecotropic viral integration site 1* (*EVI1*) gene, which in healthy individuals is transcribed in hematopoietic stem and progenitor cells but not in mature blood cells [1], [2], [3], [4], is associated with a poor prognosis in myeloid leukemias [2], [5], [6], [7], [8], [9], [10], [11], [12], [13]. *EVI1* overexpression also correlates with shorter survival in some solid tumors like ovarian carcinoma [14] and estrogen receptor negative breast cancer [15]. Despite the well documented importance of *EVI1* in predicting and most likely causing chemotherapy resistance in human malignant diseases, knowledge about its mechanism of action is limited. EVI1 is thought to act mainly as a transcription factor, and a recent publication provided a comprehensive overview of genes that are directly regulated by EVI1 in ovarian carcinoma cells [16]. However, only a small number of EVI1 target genes have been functionally characterized so far. Among them, the *phosphatase and tensin homolog* (*PTEN*) gene appears to be of particular interest, as direct repression of *PTEN* by EVI1 lead to activation of the AKT/mTOR pathway in murine bone marrow cells, and rapamycin prolonged survival of mice with *EVI1* overexpressing leukemias [17]. Increased AKT signalling is frequently observed in acute myeloid leukemia (AML) and has been reported to be associated with poor outcome [18], [19]. However, recent results indicate that in fact the opposite may be true [20], [21], [22], so that it is presently unclear to which extent activation of the AKT pathway can explain the poor prognosis associated with *EVI1* overexpression in AML.

Nonetheless, enhancement of AKT signalling has also been implicated in *EVI1* mediated resistance to transforming growth factor beta (TGF-β) and taxol induced apoptosis in colon cancer cells [23]. *EVI1* also increased cellular resistance towards ultraviolet (UV) light [24], tumor necrosis factor α (TNF-α) [24], and interferon α (IFN-α) [25]. In addition to activation of the AKT pathway [23], inhibition of the proapoptotic jun N-terminal kinase (JNK) [24] and repression of the induction of the *promyelocytic leukemia* (*PML*) gene [25] have been reported as possible mechanisms for *EVI1* induced apoptosis resistance. Thus, several studies have addressed the role of *EVI1* in protecting cells from apoptotic stimuli, yet little information is available regarding a possible effect of *EVI1* on cellular resistance to drugs used in the therapy of AML.

The protein product of the *CDKN1A/p21/WAF* gene, p21, is a 165 amino acid protein best known for its role in mediating p53 dependent cell cycle arrest [26]. In agreement with a tumor suppressive role of this gene, many human cancers exhibit low levels of p21 protein, and experimental ablation of *CDKN1A* promotes tumor formation in mice [26]. On the other hand, *CDKN1A* may also be overexpressed in human malignancies, and this molecular alteration is associated with therapy resistance and poor survival [26], [27], [28]. Accordingly, p21 protects cells from apoptosis induced by DNA damaging agents and other kinds of stress *in vitro* and *in vivo* [26], [27], [29], [30], [31], [32], [33], [34], [35], [36], [37]. Preexisting p21 may play a role in increased apoptosis resistance in some cases [27], [28], [38]. In addition, many of the stimuli from which it protects themselves induce p21 [26], [29], [31], [32], [33], [34], [37], [39]. The regulation of *CDKN1A/p21/WAF* is complex and involves a number of transcription factors in addition to p53, as well as posttranscriptional mechanisms like mRNA-miRNA interactions and phosphorylation, which, among others, affects its subcellular location [26], [27], [37], [39]. The antiapoptotic function of p21 has often been associated with its residence in the cytoplasm [27], [34], where it inhibits proapoptotic molecules like JNK and caspases [26]. However, nuclear activities of p21 also contribute to its survival promoting activity: apart from an indirect effect through cell cycle inhibition, its ability to directly bind to and inhibit the activity of transcription factors like E2F1 and MYC plays a role [26], [34].

In AML, overexpression of p21 protein relative to healthy controls was found in 17/100 patient samples, and was associated with worse complete response rates and shorter overall survival [28]. Agents used for chemotherapy in AML like etoposide and anthracyclines induced *CDKN1A/p21/WAF* in a variety of cell types, including hematopoietic cells [29], [31], [32], [33], [34], [37], [39], and p21 protected hematopoietic cells from apoptosis [29], [30], [31], [33], [35], [36]. In addition to cytostatic agents, leukemogenic oncoproteins like BCR-ABL, PML-RARα, AML1-ETO, and FLT3-ITD induced p21 in myeloid cells [35], [40], [41]. Experimental ablation of p21 in *PML-RARα* and *AML1-ETO* expressing hematopoietic cells increased the number of double strand breaks induced by these fusion proteins and reduced their leukemogenicity, suggesting that protection from excessive oncogene induced DNA damage by p21 was essential for the maintenance of leukemia stem cells in this context [40].

In the present report we describe an *in vitro* model that recapitulates the resistance of *EVI1* overexpressing cells to drugs used in the chemotherapy of AML, and show that this effect may in part be mediated by upregulation of *CDKN1A/p21/WAF*.

## Materials and Methods

### Ethics Statement

Animal experiments were approved by the ethics committee of the Medical University of Vienna and the Bundesministerium für Wissenschaft und Forschung Ref. II/10b (Gentechnik und Tierversuche), application Nr. BMWF-66.009/0095-II/10b/1010, and were carried out according to the Austrian and FELASA guidelines for animal care and protection in order to minimize distress for the animals. Mice were sacrificed by cervical dislocation.

### Cell Culture, Retroviral Vectors, and Infections

The human hematopoietic cell lines U937 [42], HNT-34 [43], and HL-60 [44] were cultured in RPMI 1640 (Life Technologies, Carlsbad, CA, USA) containing 10% fetal bovine serum (FBS; Life Technologies) and 1% Penicillin/Stretopmycin/Glutamine (PSG; Life Technologies) in a humidified incubator at 37°C and 5% CO_2_. Phoenix-gp cells (http://www.stanford.edu/group/nolan/retroviral_systems/phx.html) were cultured in DMEM (Life Technologies) with 10% FBS and 1% PSG at 37°C and 5% CO_2_.

The full length cDNA of the human *EVI1* gene, which codes for a 1051 amino acid protein, was cloned into the BamHI and EcoRI sites of the retroviral vector pBMN_IRES-eGFP (Addgene, Cambridge, MA, USA) to yield pBMN_EVI1-IRES-eGFP using standard molecular biology techniques. For doxycycline inducible expression of human *EVI1*, a codon optimized version of its cDNA (Geneart, Regensburg, Germany) was inserted into the BamH1 and AsiSI sites of pCCL.SIN.cPPT.TRE.IRES.eGFP.wPRE (F.H., unpublished results) and verified by sequencing. The human *CDKN1A* gene was amplified from cDNA from etoposide treated U937_EVI1 cells using primers CDKN1A_fwd (5′-CACGGATCCGAGGCGCCATGTCAGAAC-3′) and CDKN1A_rev (5′-CTGACTCGAGGGATTAGGGCTTCCTCTTGG-3′) and Phusion High Fidelity Polymerase (New England Biolabs, Ipswich, MA, USA). It was cloned into the retroviral vector pMIA-II_IRES-Ametrine using the BamHI and XhoI sites to yield pMIA-II_CDKN1A-IRES-Ametrine. The identity of the insert was confirmed by DNA sequence analysis. pMIA-II_IRES-Ametrine was a kind gift from Dr. Dario Vignali of the St. Jude Children’s Research Hospital, Memphis, Tennessee. Ametrine is a fluorescence marker with spectral properties similar to those of Cyan Fluorescent Protein (CFP), but brighter.

Retroviral particles were generated and U937 and their derivative cell lines infected using standard procedures [45]. Three days later, cells were sorted for enhanced Green Fluorescent Protein (eGFP) or Ametrine positivity on a FACS Aria (Becton Dickinson, Franklin Lakes, NJ, USA). Cell line authentication by short tandem repeat profiling (performed by Ingenetix, Vienna, Austria) confirmed the identity of pBMN_EVI1-IRES-eGFP and pBMN_IRES-eGFP infected cells (U937_EVI1 and U937_vec cells, respectively) as U937 derivatives. To generate HL-60 cells expressing *EVI1* in a doxycycline inducible manner, parental HL-60 cells were first infected with pLVX-Tet-On Advanced (Clontech, Mountain View, CA, USA) using previously described methodology [46]. After selection with 1 mg/ml G418 (Clontech), rtTA expressing cells were transduced with pCCL.SIN.cPPT.TRE.EVI.IRES.eGFP.wPRE or empty vector as a control, yielding HL-60_EVI1 and HL-60_vec cells, respectively. Transduced cultures were induced for 48 h with 2 µg/ml doxycycline (Clontech) and sorted for eGFP positivity. Doxycycline inducible gene expression was verified by FACS and immunoblot analysis.

### Sequence Analysis

The *CDKN1A* cDNA in pMIA-II_IRES-Ametrine was sequenced using the primers used for PCR amplification. Sequence analysis of the integrated exogenous *EVI1* gene in U937_EVI1 cells was performed after amplification of 5 overlapping gene segments covering the entire *EVI1* coding sequence from genomic DNA isolated from U937_EVI1 cells after several weeks in culture. Primers used for amplification of *EVI1* are shown in Table 1. Capillary sequencing was performed by Eurofins-MWG-Operon (Ebersberg, Germany).

**Table 1:** Primers used for amplification and sequence analysis of the integrated, vector borne *EVI1* gene from U937_EVI1 cells.

Primer name	sequence (5′–3′)
F1-F*	GCTTGGATACACGCCGC
F1-R	GGTCACTGCCACTTGGTGTA
F2-F	GAGGTTTTGTGAGGGCAAGA
F2-R	ACCCACTCCTTTCTTTATGGACC
F3-F	ACCCACACAGGAGAGCAGCC
F3-R	TACGTGGCTTATGGACTGGATAGCAC
F4-F	AGCAACGTCGAATCAAGACCTGCTTCAGAT
F4-R	CGGACTAGTTGCTGTACCGGACATGTTCCC
F5-F	TGCTCTAGATCATATAAGGCA
F5-R*	GGAGAGGGGCGGAATTTAC

* Primers F1-F and F5-R match the vector, rather than the *EVI1*, sequence.

### Integration Site Analysis by LAM-PCR

500 ng genomic DNA, isolated from U937_vec and U937_EVI1 cells either shortly after infection and sorting or after 12–15 weeks in culture, were digested with Tsp509I or HinPI and used for the analysis of integration sites by 5′-LTR-mediated linear amplification mediated (LAM)-PCR as previously described [47]. Briefly, linear PCR was performed using vector specific 5′-biotinylated primers LTRa bio (5′-TGCTTACCACAGATATCCTG-3′) and LTRb bio (5′-ATCCTGTTTGGCCCATATTC-3′). For the first and second exponential PCRs, vector specific primers LTR II bio (5′- GACCTTGATCTGAACTTCTC-3′) and LTR III bio (5′-TTCCATGCCTTGCAAAATGGC-3′) were used in combination with linker cassette specific primers LC I (5′-GACCCGGGAGATCTGAATTC-3′) and LC II (5′-GATCTGAATTCAGTGGCACAG-3′). 5% of the LAM-PCR amplicons were separated on a Spreadex high-resolution gel (Elchrom Scientific, Cham, Switzerland).

For high throughput pyrosequencing (GS FLX, Roche Diagnostics, Risch, Switzerland), 40 ng per sample were prepared according to the manufacturer’s protocol. Approximately 26.000 sequence tags were obtained from each sample. They were mapped to the human genome using the UCSC blast-like alignment tool (BLAT) (GRCh37/hg19, Feb 2009, available at http://www.genome.ucsc.edu/cgi-bin/hgBlat).

### Microarray Analyses

RNA for microarray analyses was extracted from two independent replicate cultures of U937_vec and U937_EVI1 cells treated or not treated with 400 nM etoposide for 48 h using the RNeasy Plus Mini kit (Qiagen, Hilden, Germany). RNA quality control, labelling, hybridization to Human Gene 1.1 ST arrays (Affymetrix, Santa Clara, CA, USA), and primary data analysis using the Affymetrix RMA algorithm were performed at the Center of Excellence for Fluorescent Bioanalytics (KFB; Regensburg, Germany). Genes whose expression changed at least twofold in response to *EVI1* (in the absence of etoposide) or to etoposide (in the absence of *EVI1*) in both replicate experiments were considered as regulated by the respective stimulus.

### RNA Extraction, Reverse Transcription, and Real Time Quantitative Reverse Transcriptase Polymerase Chain Reaction (qRT-PCR)

Total RNA for qRT-PCR was extracted using Trizol (Life Technologies, Carlsbad, CA, USA) and reverse transcribed using random hexamer primers (Life Technologies) and M-MLV reverse transcriptase (Life Technologies) according to the manufacturer’s instructions. qRT-PCR was carried out in a Step One Plus Real Time PCR system (Applied Biosystems, Life Technologies). Levels of *CDKN1A* and of the housekeeping gene *β-2-microglobulin (B2M)* were determined using the corresponding TaqMan Gene Expression Assays (*CDKN1A*: Hs00355782_m1*, *B2M*: 4333766F) and the TaqMan Gene Expression Mastermix (all from Applied Biosystems, Life Technologies). All assays were carried out in triplicate. Expression values for the gene of interest relative to the housekeeping gene and to a reference value were determined using the ΔΔC_T_ method [48].

### Preparation of Protein Extracts and Immunoblot Analysis

To prepare whole cell extracts for immunoblot analysis, exponentially growing cells were left untreated or treated with 400 nM etoposide for 48 h, pelleted, and boiled in SDS loading buffer (62.5 mM Tris-HCl pH 6.8, 5 mM EDTA, 2% (w/v) SDS, 10% Glycerol, 5% β-mercaptoethanol, and bromophenol blue). Cytoplasmatic extracts were prepared by lysis of the plasma membrane with a buffer containing 300 mM sucrose, 10 mM Hepes pH 7.9, 10 mM KCl, 0.1 mM EDTA, 0.1 mM EGTA, 1 mM DTT, 0.75 mM Spermidine, 0.15 mM Spermine, 0.1% NP-40, and protease inhibitors. The remaining nuclei were then extracted with a buffer containing 20 mM Hepes pH 7.9, 420 mM NaCl, 25% glycerol, 1 mM EDTA, 1 mM EGTA, 1 mM DTT, and protease inhibitors. An appropriate amount of 4× SDS loading buffer was added, and samples were boiled. Polyacrylamide gel electrophoresis, tank blotting onto Bio Trace PVDF membranes (Pall Corporation, Port Washington, NY, USA), and antibody hybridizations were performed using standard procedures. Primary antibodies directed against EVI1 (rabbit mAb anti-EVI1 C50E12, Cell Signaling Technology, Danvers, MA, USA) and p21 (rabbit mAb anti-p21Waf1/Cip1 12D1, Cell Signaling Technology) were used at a 1∶1000 dilution; the antibody against the nuclear marker protein RCC1 (mouse mAb anti-RCC1 E-6, Santa Cruz Biotechnology, Santa Cruz, CA, USA) was used at a dilution of 1∶500; and the antibody against the housekeeping gene β-tubulin (mouse mAb anti-β-tubulin clone TUB 2.1, Sigma-Aldrich, Seelze, Germany) was used at a dilution of 1∶2.500. Horseradish peroxidase conjugated goat anti-mouse and goat anti-rabbit secondary antibodies (Jackson ImmunoResearch Labaratories, West Grove, PA, USA) were used at dilutions of 1∶50.000–1∶75.000 and detected using the SuperSignal West Pico Chemiluminescent Substrate (Pierce, Thermo Fisher Scientific, Rockford, IL, USA) or, for faint signals, the Super Signal West Femto kit (Pierce).

### Intranuclear *EVI1* Staining

5×10^5^ cells per sample were fixed with 2% formaldehyde in phosphate buffered saline (PBS) for 10 min at 37°C, and permeabilized by dropwise addition of a tenfold volume of ice cold methanol, followed by a 30 min incubation on ice or storage at −20°C for up to several weeks. Cells were blocked for 15 min with 1% BSA (Sigma-Aldrich) and Beriglobin (1∶80, CSL-Behring) in PBS, and incubated for 1 h with 11 ng EVI1 antibody (C50E12, Cell Signaling Technology) or isotype control (DA1E, rabbit IgG, Cell Signaling Technology) in a total volume of 60 µl. Secondary antibody incubation was for 30 min with 0.02 ng Alexa Fluor 647 F(ab′)2 fragment of goat anti rabbit IgG(H+L) (Life Technologies) in a total volume of 60 µl. FACS analysis was performed on a FACS LSR Fortessa (Becton Dickinson) using FACSDiva Software.

### Analyses of Cell Cycle Distribution, Differentiation, Viability, and Apoptosis

For cell cycle analyses, cells were adjusted to a density of 400 cells/µl. On the next day, they were washed with PBS and incubated for 5 min in ice cold 0.5 M citrate/0.5% Tween-20. Cell membranes were disrupted mechanically before nuclei were pelleted and resuspended in PBS containing 100 µg/ml RNase A (Sigma-Aldrich) and 50 µg/ml propidium iodide (PI; Sigma-Aldrich). Nuclear DNA content was determined on a FACS Calibur (Becton Dickinson) or a FACS LSR Fortessa using ModFit software (Verity Software House) for data analysis.

To induce differentiation of U937 derivative cell lines, 12-O-tetradecanoylphorbol 13-acetate (TPA; Sigma-Aldrich) at a final concentration of 50 ng/ml was added to cells that had been adjusted to a density of 100 cells/µl. Control cultures were treated with an equivalent amount of solvent (ethanol). 5 days later, cells were stained with a phycoerythrin (PE) conjugated antibody against the monocyte/macrophage specific cell surface marker CD11b (anti-CD11b antibody D12, Becton Dickinson). Flow cytometric analyses were carried out on a FACS Calibur. The percentage of CD11b positive cells relative to the isotype control was used as a measure of differentiation. For morphological analysis of differentiation, cytospin preparations were stained according to a modified Wright technique.

To determine cellular viability/metabolic activity or apoptosis, cells were seeded into white walled 96-well plates (Greiner Bio-One, Kremsmünster, Austria) to a final density of 50 cells/µl, and etoposide or daunorubicin were applied at the indicated concentrations. For HL-60 derivative cell lines, 0.5 µg/ml doxycycline was added to the cells 24 h prior to the addition of daunorubicin. 48 h after the addition of cytotoxic drugs, metabolic activity or the activities of caspases 3 and 7 were determined using the Cell Titer Glo or Caspase-Glo 3/7 assays, respectively (both from Promega, Madison, WI, USA). Nuclear morphology after a 48 h incubation in the presence or absence of 400 nM etoposide was assessed by spinning cells onto microscopic slides and staining them with 1 µg/ml 4′,6-Diamidin-2-phenylindol (DAPI; Roche). Coverslips were mounted using Permafluor Mountant (Labvision, Thermo Fisher Scientific). Slides were inspected under a LSM 700 microscope (Zeiss, Oberkochen, Germany) and intact and apoptotic nuclei were counted by an observer blind to the identity of the samples. Nuclei were considered apoptotic if they were partially fragmented or very large and diffuse. At least 200 nuclei were counted per sample. For annexin V/propidium iodide staining, HL-60_EVI1 and HL-60_vec cells were seeded to a density of 50 cells/µl and treated with doxycycline for 24 h prior to the addition of 60 nM daunorubicin. After another 48 h, cells were stained with AnnexinV-APC (Becton Dickinson Biosciences; 1∶200) and propidium iodide (AppliChem), and analyzed on a FACS BD LSR II (Becton Dickinson) using FACS Diva Software (Becton Dickinson Biosciences).

### Xenograft Experiments, Histological Analysis, and Immunohistochemistry

Six to eight week old female CB-17 scid/scid (SCID) mice were purchased from Harlan Laboratories (San Pietro al Natisone, Italy). The animals were kept in a pathogen-free environment and all procedures were performed in a laminar airflow cabinet.

5×10^6^ U937_vec or U937_EVI1 cells, resuspended in 50 µl of serum free RPMI 1640 medium, were injected subcutaneously into the right flank of each mouse. Animals were controlled every day and tumor size was assessed regularly by caliper measurement. Tumor volume was calculated using the formula: (length × width^2^)/2. At experiment termination, mice were dissected and tumor tissue was processed for standard histological examinations. For statistical analysis, adjusted areas under curves (aAUCs) were calculated, assuming no tumor volume at day 3, and compared by nonparametric bootstrap inference [49].

Immunohistochemistry (IHC) was performed using standard procedures. 4 µm sections from xenograft tumor blocks were deparaffinized and rehydrated, and either stained by hematoxylin and eosin for histological confirmation of the presence of invasive carcinoma, or subjected to IHC. For IHC, tissue sections were heated for 10 min in 10 mM citrate buffer (pH 6.0) in a pressure cooker for epitope retrieval, and then incubated for 60 min at room temperature with rabbit mAb anti-EVI1 (clone C50E10, Cell Signaling Technology; dilution 1∶200) or rabbit mAb anti-CD11b (clone EPR1344, Abcam; dilution 1∶100). Antibody binding was detected by means of the UltraVision LP detection system according to the manufacturer’s recommendations (Lab Vision, Thermo Fisher Scientific). Color development was performed by 3–3′-diaminobenzidine and counterstaining by hematoxylin.

## Results

### Establishment and Characterization of an *in vitro* Model for Human Myeloid Leukemias Constitutively Overexpressing *EVI1*

**Figure 1:**
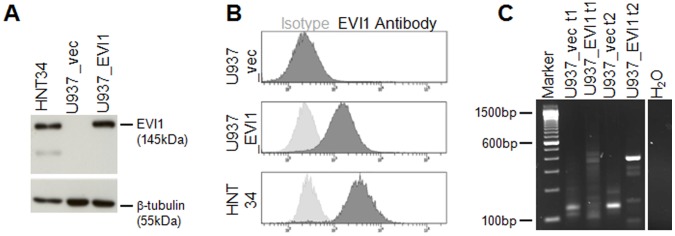
Establishment and characterization of a human myeloid cell line constitutively overexpressing *EVI1*. A) Immunoblot analysis for the detection of EVI1 in U937_vec and U937_EVI1 cells. HNT-34 cells, which express EVI1 due to a rearrangement of chromosome band 3q26 [43], were included for comparison. Hybridization with a β-tubulin antibody was used as a loading control. B) Intracellular FACS staining for detection of EVI1 in U937_vec, U937_EVI1, and HNT-34 cells. Dark grey histogram curves, EVI1 antibody; light grey histogram curves, isotype control. C) High-resolution gel analysis of LAM-PCR amplicons obtained from Tsp509I digested genomic DNA from U937_vec and U937_EVI1 cells. DNA was isolated shortly after infection and sorting (t1) as well as after another 12–15 weeks in culture (t2).

In order to establish an *in vitro* model for human myeloid leukemias constitutively overexpressing *EVI1*, the human myeloid cell line U937 was infected with a retroviral vector containing the full length human *EVI1* cDNA (pBMN_EVI1-IRES-eGFP), or with empty vector (pBMN_IRES-eGFP) as a control. FACS sorting for eGFP positivity yielded the cell lines U937_EVI1 and U937_vec, respectively. Immunoblot and FACS analyses confirmed overexpression of the EVI1 protein in the bulk U937_EVI1 population and at the single cell level, respectively (Fig. 1A, B). EVI1 protein levels in U937_EVI1 cells were comparable to those in HNT-34 cells, which overexpress endogenous EVI1 due to a rearrangement of chromosome band 3q26 [43] (Fig. 1A, B), and were stable over culture periods of several weeks and during freezing and thawing of cells (data not shown). Amplification and sequence analysis of the exogenous *EVI1* gene from U937_EVI1 cells confirmed that it was intact and did not contain any mutations.

To investigate whether a cooperating gene might be activated by the retroviral vector, integration site analysis was performed by LAM PCR. Shortly after being sorted for eGFP positivity, U937_vec and U937_EVI1 cells contained 25 and 10 uniquely mappable integration sites, respectively. After another 12–15 weeks of total culture time, interrupted by two freeze-thaws, the number of integration sites was reduced to 15 for U937_vec and five for U937_EVI1 cells (Fig. 1C, and data not shown). At that time, both lines contained predominant integration sites: in U937_vec cells, 79% of these mapped near the *dermatan sulfate epimerase* (*DSE*) gene in chromosome band 6q22, and 99% of the integration sites in U937_EVI1 cells were located in the vicinity of the *ubiquinol-cytochrome c reductase-Rieske iron-sulfur polypeptide 1* (*UQCRFS1*) gene in 19q12-13. However, according to gene expression microarray analysis neither of the two genes was differentially expressed between the two cell lines. Also, no other gene in band 2 of chromosome arm 6q or in 19q was upregulated by more than 3-fold in U937_vec or U937_EVI1 cells compared to the respective other cell line, indicating that retroviral integration did not cause activation of adjacent genes.

### Effects of *EVI1* on Proliferation of U937 Cells *in vitro* and *in vivo*

**Figure 2:**
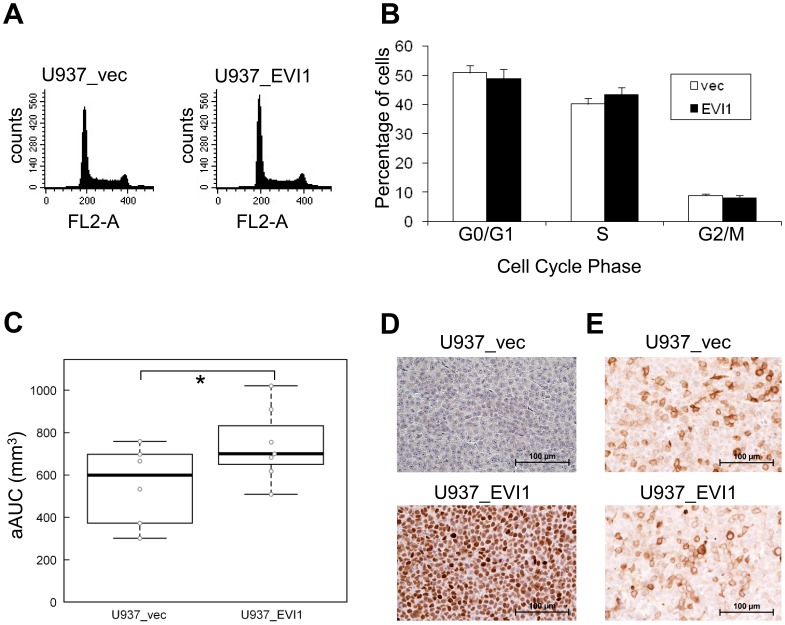
Effects of *EVI1* on growth dynamics *in vitro* and *in vivo*. A) Examples of cell cycle profiles of U937_vec and U937_EVI1 cells. B) Cell cycle distribution of U937_vec and U937_EVI1 cells. Shown are the means+standard errors of the mean (SEM) from 3 independent experiments. None of the differences between the two cell lines are statistically significant (Student’s t-test). C) Tumor growth after subcutaneous injection of U937_vec and U937_EVI1 cells into the flanks of CB-17 scid/scid mice. The adjusted area under the curve (aAUC) was calculated for each tumor, and the two groups of tumors were compared by nonparametric bootstrap inference. *, p<0.05. D) Immunohistochemical (IHC) staining showing persistent expression of EVI1 in U937_EVI1 derived tumor xenografts. E) IHC revealing the presence of similar proportions of CD11b positive cells in U937_EVI1 and U937_vec derived tumors.

The growth rates of U937_vec and U937_EVI1 cells did not appear grossly different during regular passaging. To determine a possible effect of *EVI1* on cell cycling with more precision, nuclei from exponentially growing *EVI1* overexpressing and control cells were stained with propidium iodide and subjected to FACS analysis. As shown in Fig. 2A and B, the cell cycle profiles of both lines differed only marginally, if at all.

To investigate the consequences of *EVI1* overexpression *in vivo*, CB-17 scid/scid mice were injected subcutaneously with U937_vec and U937_EVI1 cells. U937_EVI1 tumors grew significantly more rapidly than tumors derived from U937_vec cells (Fig. 2C), and all animals with *EVI1* overexpressing tumors had to be sacrificed by day 17 of the experiment versus day 22 for mice with control tumors. Immunohistochemical analysis confirmed that EVI1 protein was persistently expressed at high levels in the U937_EVI1 derived tumors (Fig. 2D). Staining with an antibody against the early myeloid differentiation marker CD11b revealed the presence of some CD11b positive cells in, but no marked differences between, U937_EVI1 and U937_vec derived tumors (Fig. 2E). In summary, even though *EVI1* had only minimally enhancing effects on cellular proliferation *in vitro*, it greatly enhanced growth and aggressiveness of tumors *in vivo*.

### *EVI1* Interferes with Differentiation of U937 Cells

**Figure 3:**
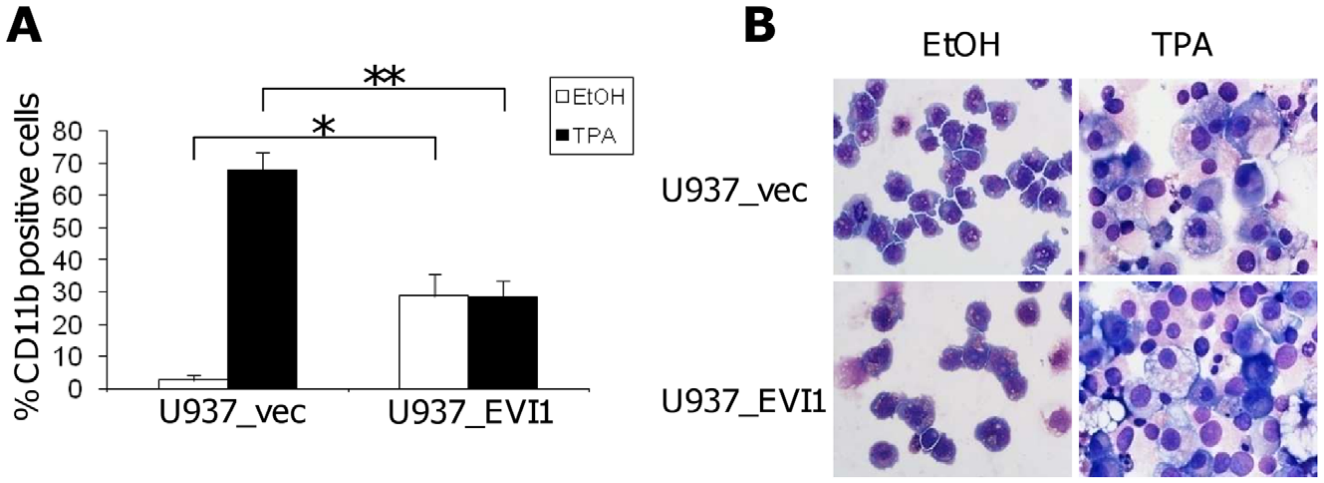
*EVI1* inhibits phorbol ester (TPA) induced differentiation of U937 cells *in vitro*. A) Percentage of CD11b positive U937_vec and U937_EVI1 cells after incubation with TPA (black bars) or solvent (ethanol; white bars) for five days. Shown are the means+SEMs from 3 independent experiments. *, p<0.05; **, p<0.01 (paired Student’s t-test). B) Morphology of Wright stained U937_vec and U937_EVI1 cells after incubation with TPA or vehicle for five days. Original magnification is 600-fold.

We next asked whether overexpression of *EVI1* would affect the differentiation of U937 cells in response to appropriate inducers *in vitro*. U937_vec and U937_EVI1 cells were cultured in the absence or presence of TPA for five days and analyzed for morphological alterations as well as changes in the cell surface expression of CD11b. As expected, the vast majority of the U937_vec cells was CD11b negative in the absence of TPA, but about 70% became positive for this marker under differentiation conditions (Fig. 3A). In contrast, 30% of the U937_EVI1 cells stained positive for CD11b even in the absence of TPA, but this percentage did not increase further in the presence of the differentiation agent (Fig. 3A). Morphological analysis revealed that both cell lines showed mainly immature characteristics in the absence of TPA (Fig. 3B). In the presence of TPA, U937_vec cells differentiated along the monocytic lineage. U937_EVI1 cells also differentiated to a certain degree, but more inhomogeneously and with more cells remaining in an immature state (Fig. 3B).

### *EVI1* Enhances Resistance to Cytostatic Drugs used in AML Therapy

**Figure 4:**
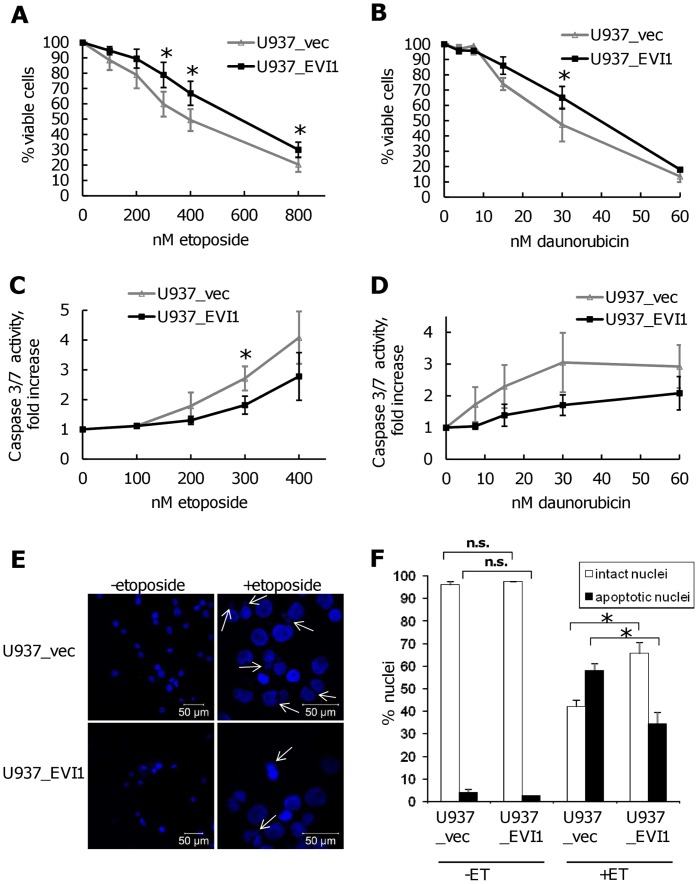
Overexpression of *EVI1* decreases the sensitivity of U937 cells to drugs used in AML therapy. A–D) U937_vec and U937_EVI1 cells were treated with the indicated concentrations of etoposide (A, C) or daunorubicin (B, D) for 48 h. Cellular viability/metabolic activity was determined based on ATP content (A, B), and apoptosis was measured via caspase 3/7 activity (C, D). Data points represent the mean +/− SEM from at least three independent experiments. *, p<0.05 (paired Student’s t-test). E) Nuclear morphology of U937_vec and U937_EVI1 cells after treatment with or without 400 nM etoposide for 48 h. Apoptotic nuclei are marked by arrows. Please note difference in scale between etoposide and control treated cells. F) Quantitative assessment of nuclear morphology. Nuclei prepared as in E were counted as ‘intact’ or ‘apoptotic’ (see Methods) by an observer blinded to the identity of the samples. Data points represent the mean+SEM from 3 independent experiments. **, p<0.01; n.s., not significant (paired Student’s t-test).

Because *EVI1* overexpression is associated with poor responsiveness to chemotherapy in AML, we next asked whether experimental expression of *EVI1* would render U937 cells more resistant to agents routinely used to treat this disease. U937_vec and U937_EVI1 cells were exposed to various doses of etoposide for 48 h and their metabolic activity was measured via an ATP-dependent luminescence reaction. Indeed, cells overexpressing *EVI1* were more resistant to treatment with this agent than their empty vector infected counterparts (Fig. 4A). A similar effect was observed with daunorubicin (Fig. 4B). These results were confirmed using a luminescence based assay measuring the activity of the executioner caspases 3 and 7 (Fig. 4C, D), as well as by enumeration of apoptotic nuclei after staining with DAPI (Fig. 4E, F). The latter experiment also showed that the rates of basal apoptosis were comparable between U937_vec and U937_EVI1 cells.

**Figure 5:**
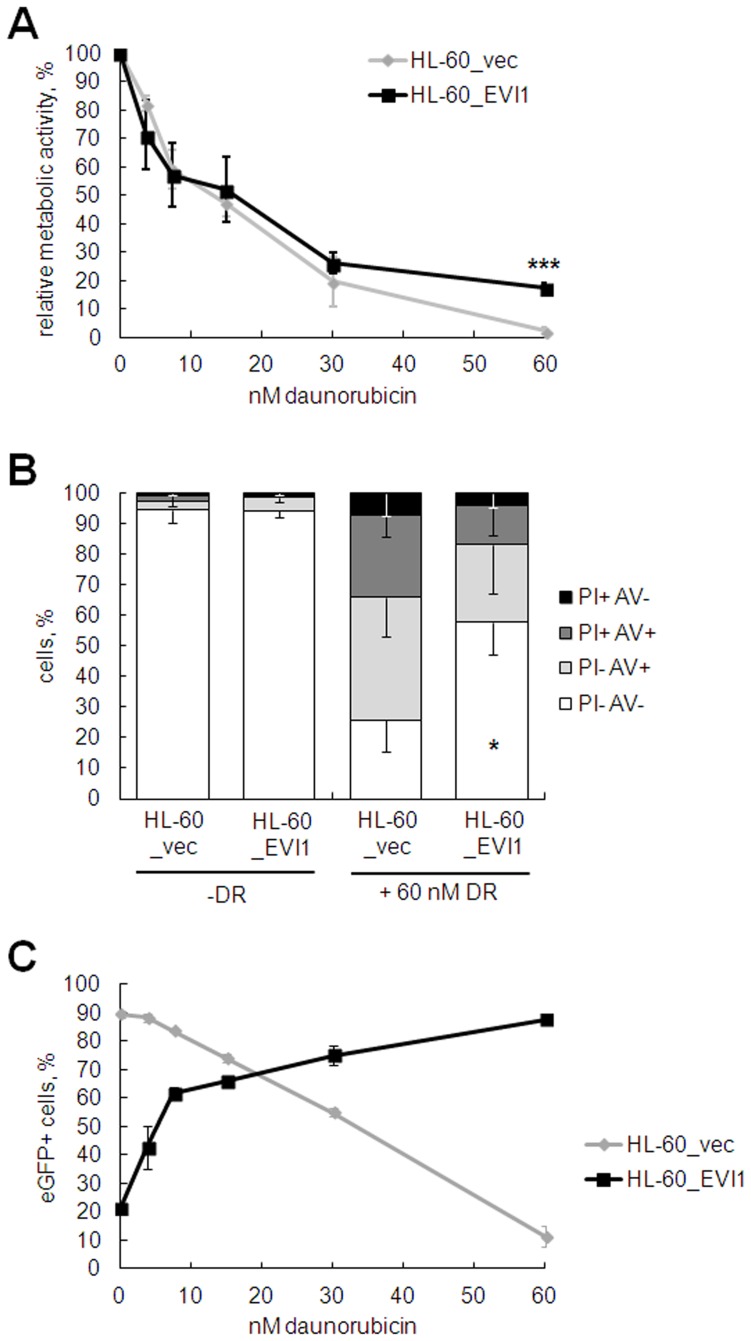
Inducible expression of *EVI1* decreases the sensitivity of HL-60 cells to daunorubicin. HL-60_EVI1 and HL-60_vec cells were incubated with 2 µg/ml doxycycline for 24 h prior to addition of daunorubicin (DR) at the indicated concentrations for another 48 h. A) Metabolic activity was measured based on ATP content. B) The proportions of viable, apoptotic, and dead cells were determined via the annexinV (AV)/propidium iodide (PI) assay. Double negative cells were considered viable, PI- AV+ cells apoptotic, and PI+ cells dead. C) Proportion of eGFP positive (eGFP+) cells in HL-60_EVI1 and HL-60_vec cells treated with various doses of daunorubicin. Data points in all panels represent the mean +/− standard deviation (SD) from three independent biological replicate experiments. *, p<0.05; ***, p<0.001 (paired Student’s t-test).

To corroborate these findings in an independent cell culture model, HL-60_EVI1, a human myeloid cell line engineered to express *EVI1* in an inducible manner, and the control cell line HL-60_vec were treated with doxycycline for 24 h before daunorubicin was added at various doses for another 48 h. Cells overexpressing *EVI1* exhibited significantly higher metabolic activity in the presence of daunorubicin than *EVI1* negative control cells (Fig. 5A). These results were confirmed by annexin V/propidium iodide staining, which revealed a significantly higher proportion of viable HL-60_EVI1 versus HL-60_vec cells in the presence of 60 nM daunorubicin (Fig. 5B). Finally, the proportion of eGFP positive HL-60_EVI1, but not HL-60_vec, cells increased with increasing doses of daunorubicin (Fig. 5C), indicating that *EVI1* expression conferred a survival advantage during treatment with the cytotoxic drug.

### *EVI1* and Etoposide Alter the Expression of Overlapping Sets of Genes

**Figure 6:**
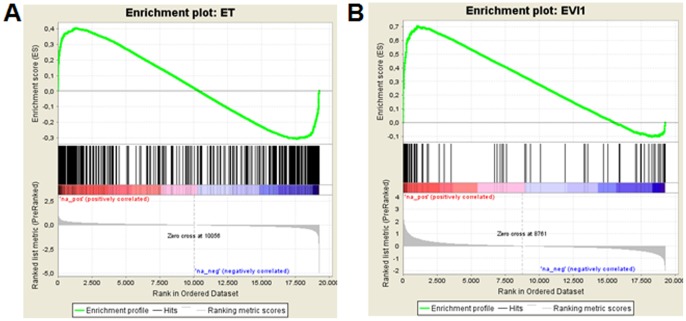
*EVI1* and etoposide regulate overlapping sets of genes. cRNAs from two independent cultures of U937_vec and U937_EVI1 cells treated or not treated with 400 nM etoposide for 48 h were hybridized onto Affymetrix ST1.1 arrays. Only genes deregulated at least 2-fold in both experiments were considered as differentially expressed. Gene set enrichment analysis (GSEA) [68] was performed to evaluate concordant differences of A) etoposide regulated genes within the gene expression list ranked by average log2-fold changes between U937_EVI1 and U937_vec cells, and B) *EVI1* regulated genes within the gene expression list ranked by average log2-fold changes between etoposide treated and untreated U937_vec cells. The normalized enrichment scores were 1.9 in A) and 2.2 in B); the p-values and the q-values of the false discovery rate were 0.0 in both analyses.

In order to gain an understanding of the molecular mechanisms mediating the *EVI1* induced resistance towards chemotherapeutic drugs, RNA was extracted from U937_vec and U937_EVI1 cells after 48 h of incubation in the absence or presence of etoposide, and hybridized to human gene 1.1 ST arrays. This experiment was performed in duplicate, and only genes induced or repressed at least 2-fold in both experiments were considered as regulated by the respective condition. According to this criterion, 77 unique genes were regulated by *EVI1* (23 induced and 54 repressed), and 419 unique genes were regulated by etoposide (327 induced and 92 repressed). 26 genes were regulated by both *EVI1* and etoposide: 14 were upregulated and 4 were downregulated by both conditions, and 8 genes were induced by etoposide and repressed by *EVI1* (Table S1). Thus, while only 0.4% of the 19255 genes on the 1.1 ST array with a current RefSeq annotation were regulated by *EVI1*, 6.2% of the etoposide regulated genes were also regulated by *EVI1* (15.5-fold enrichment, p = 9.9×10^−24^, Fisher’s exact test). Conversely, 2.2% of all genes, but 33.8% of the *EVI1* regulated genes were regulated by etoposide (15.4-fold enrichment, p = 6.0×10^−21^, Fisher’s exact test) (Fig. 6).

While our results confirm the recently reported induction of *CD52* by *EVI1* [50], *ITGA6*, another presumptive *EVI1* target gene [51], was not induced, but rather slightly downregulated in U937_EVI1 cells. Likewise, *PTEN* was not repressed by *EVI1* in our system, even if the threshold for differential expression was lowered to the 1.3-fold used in the publication reporting this relationship [17]. In search of a molecular mechanism that could explain the partial resistance of U937_EVI1 cells to chemotherapeutic drugs, we subjected genes coding for classical apoptosis regulators to particular scrutiny. However, even with a cut-off of 1.3-fold *BCL2*, *MCL1*, *BAG3*, *NOXA1*, *BID*, *BIK*, *BAX*, *BAK1*, and *XIAP* were not regulated by *EVI1*. The survival genes *BCL2A1* and *BCL2L1* were induced 1.3-fold and repressed 1.6-fold, respectively, in U937_EVI1 versus U937_vec cells. In contrast, the antiapoptotic gene *CDKN1A/p21/WAF* was strongly induced both by *EVI1* and etoposide.

### *CDKN1A/p21/WAF* Contributes to the Effects of *EVI1* on Cellular Resistance to Cytostatic Drugs

**Figure 7:**
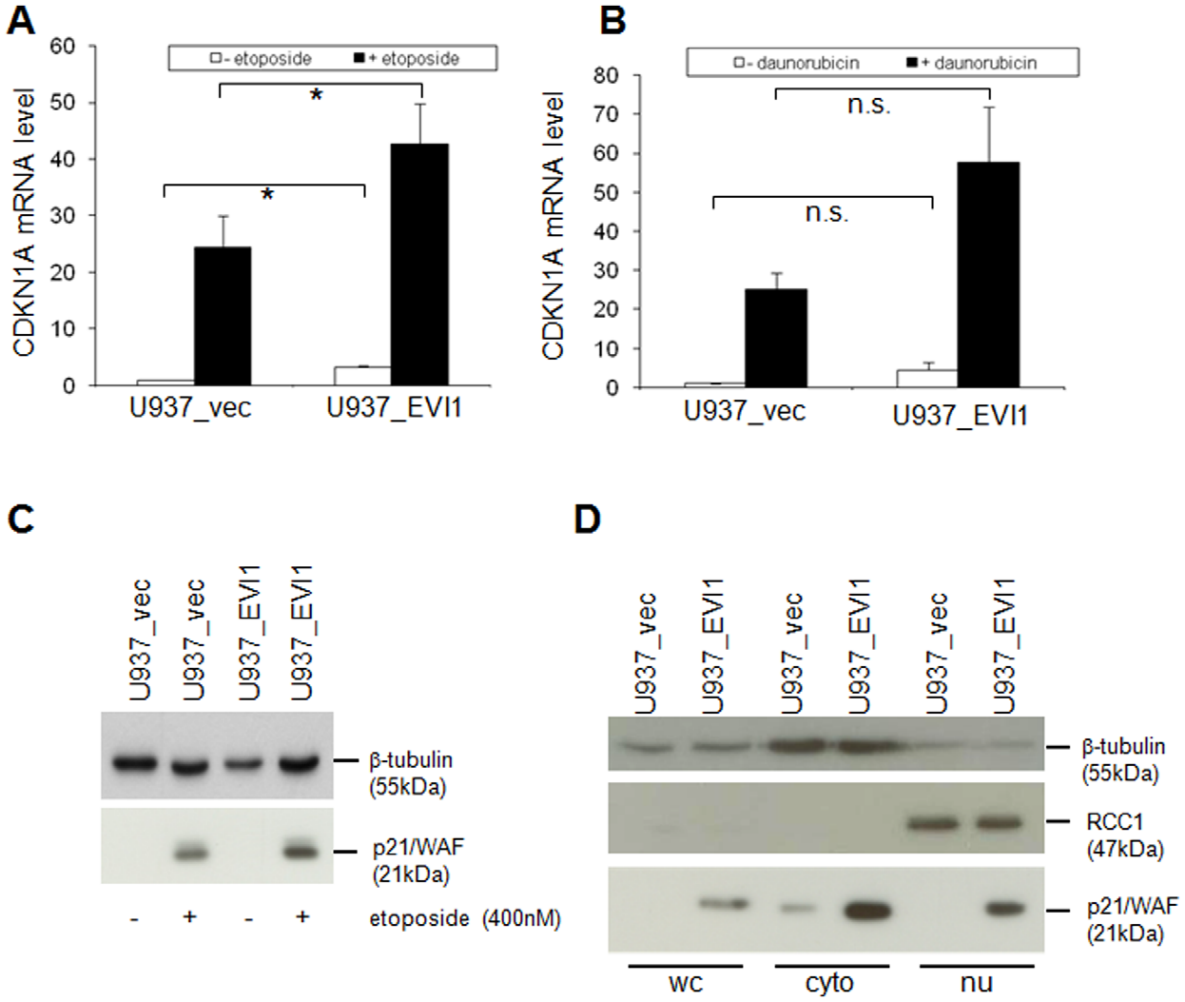
Regulation of the CDKN1A/p21/WAF mRNA and protein by *EVI1*, etoposide, and daunorubicin. A, B) qRT-PCR on RxNA from U937_vec and U937_EVI1 cells treated or not treated with 400 nM etoposide (A) or 30 nM daunorubicin (B) for 48 h. CDKN1A levels were normalized to those of the housekeeping gene B2M using the ΔΔct method [48], with untreated U937_vec cells as a calibrator. Shown are means+SEMs from 3 independent experiments. *, p<0.05; n.s., not significant (paired Student’s t-test). C) Immunoblot analysis of p21 protein in U937_vec and U937_EVI1 cells treated or not treated with 400 nM etoposide for 48 h. Hybridization with a β-tubulin antibody was used as a loading control. In the absence of etoposide, p21 is below detection level with the exposure time used. D) Immunoblot analysis of whole cell (WC), cytoplasmatic (cyto), and nuclear (nu) extracts from U937_vec and U937_EVI1 cells. The same amount of protein was loaded for cytoplasmatic and nuclear extracts, corresponding to up to twice as many cell equivalents for the latter. The cytoplasmatic protein β-tubulin and the nuclear protein RCC1 were used as loading controls.

Because *CDKN1A/p21/WAF* has been shown to mediate resistance against DNA damaging agents in a number of studies, and because it was strongly regulated by both *EVI1* and etoposide in our model system, we focussed further studies on this gene. qRT-PCR confirmed a >3-fold induction of *CDKN1A/p21/WAF* by *EVI1* and a >20-fold induction by both etoposide and daunorubicin (Fig. 7A, B). Immunoblot analysis corroborated the regulation of p21 by *EVI1* and etoposide at the protein level (Fig. 7C, D). To investigate the subcellular localization of p21 as well as a possible effect of *EVI1* on it, immunoblot analysis was performed on nuclear and cytoplasmatic extracts from U937_vec and U937_EVI1 cells. These experiments showed that the p21 protein was predominantly (but not exclusively) cytoplasmatic in both U937_vec and U937_EVI1 cells (Fig. 7D).

**Figure 8:**
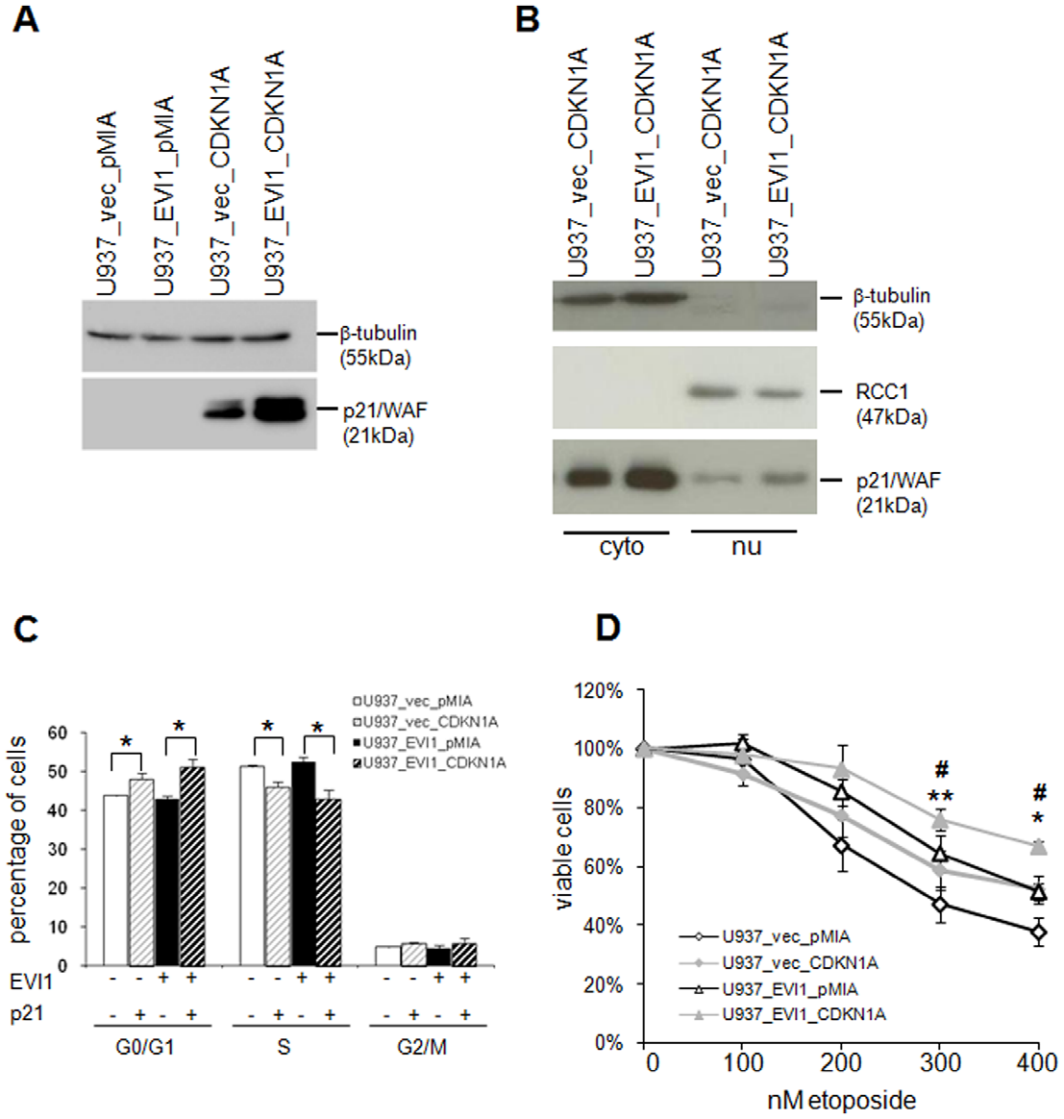
*CDKN1A/p21/WAF* overexpression partially mimics the chemotherapy resistance phenotype of *EVI1* overexpression. A) Immunoblot analysis confirming overexpressi on of p21 in U937_vec and U937_EVI1 cells infected with pMIA-II_CDKN1A-IRES-Ametrine. β-Tubulin was used as loading control. Detection of endogenous p21 would require longer exposure times. B) Immunoblot analysis of cytoplasmatic (cyto) and nuclear (nu) extracts from U937_vec and U937_EVI1 cells infected with pMIA-II_CDKN1A-IRES-Ametrine. The same amount of protein was loaded in each lane (corresponding to up to twice as many cell equivalents for nuclear versus cytoplasmatic extracts). The cytoplasmatic protein β-tubulin and the nuclear protein RCC1 were used as loading controls. C) Cell cycle distribution of *CDKN1A* overexpressing and control cells. Shown are means+SEMs from 3 independent experiments. *, p<0.05 (Student’s t-test). D) *CDKN1A* overexpression increases resistance to etoposide. Cells were treated with the indicated concentrations of etoposide for 48 h and ATP content was determined as a proxy for cellular viability. Shown are means +/− SEMs from three independent experiments. Open symbols, cells without overexpression of *CDKN1A*; closed symbols, cells overexpressing *CDKN1A*; diamonds, U937_vec derivatives; triangles, U937_EVI1 derivatives. *, p<0.05; **, p<0.01 (paired Student’s t-test; referring to the difference between the U937_vec derived cell lines); #, p≤0.05 (referring to the difference between the U937_EVI1 derived cell lines).

In order to ask whether upregulation of *CDKN1A/p21/WAF* was required for *EVI1* induced chemotherapy resistance, we attempted to knock down this gene using siRNAs, shRNAs, or antisense constructs. However, all of these experiments were unsuccessful due to a lack of efficiency and/or strong elevation of basal p21 levels under control conditions. We therefore infected U937_vec and U937_EVI1 cells with retroviral vectors containing the *CDKN1A/p21/WAF* cDNA (pMIA-II_CDKN1A-IRES-Ametrine) or the corresponding empty vector (pMIA-II_IRES-Ametrine) to investigate whether experimental expression of *CDKN1A* would mimic the phenotype caused by experimental expression of *EVI1*. Infected cells were sorted for Ametrine positivity, and overexpression of p21 was verified by immunoblot analysis (Fig. 8A). The localization of experimentally expressed p21 resembled that of endogenous p21, being mostly, but not exclusively, cytoplasmatic (Fig. 8B). FACS analysis of propidium iodide stained nuclei showed that overexpression of p21 increased and decreased the proportions of cells in G_0_/G_1_ and S phase, respectively, in both U937_vec and U937_EVI1 cells (Fig. 8C). These effects were relatively small, possibly due to the fact that exogenous p21 was mostly located in the cytoplasm. *CDKN1A/p21/WAF* overexpression also conferred partial resistance to etoposide as measured by the cell viability assay (Fig. 8D). This phenotype was present both in U937_vec and in U937_EVI1 cells, reflecting the fact that p21 was elevated above endogenous levels in both cell lines.

## Discussion

*EVI1* has been reported to affect the proliferation, differentiation, and apoptosis of hematopoietic cells, albeit not necessarily in a consistent manner [52]. For example, *EVI1* accelerated, left unaffected, or retarded the cell cycle, depending on the experimental model under investigation [51], [52], [53], [54], [55], [56], [57], [58], [59]. Species differences may play a role in that the growth of murine cells was mostly stimulated by *Evi1* [55], [56], while the proliferation of human hematopoietic cells was inhibited according to several recent reports [51], [53], [54]. Our own earlier results showed that inducible expression of *EVI1* in U937 cells strongly inhibited their proliferation [53], and prolonged culture of these cells under inducing conditions was associated with loss of *EVI1* expression (T.A. Konrad, D.H., and R.W., unpublished results). Similarly, Yamakawa et al reported that *EVI1* overexpressing U937 cells grew more slowly and accumulated in G_0_ to a higher extent than the corresponding control cell line [51]. In contrast, the U937_EVI1 cell line we describe here proliferated normally *in vitro* and sustained *EVI1* overexpression over prolonged periods of time. When grafted into SCID mice, U937_EVI1 cells even gave rise to significantly larger and faster growing tumors than the corresponding control cells. The reasons for the difference between the *in vivo* and *in vitro* growth phenotypes of U937_EVI1 cells are likely to be related to effects of the microenvironment. Together with published data, our results suggest that the effects of *EVI1* on cellular growth and proliferation are complex and highly context dependent, and their elucidation will require additional investigations in a variety of complementary model systems.

To address the possibility that the phenotypes of U937_EVI1 cells were partially or entirely due to transcriptional activation of genes by retroviral vector insertion rather than to expression of the *EVI1* gene itself, LAM-PCR was performed. Even though these experiments revealed that U937_EVI1 cells were nearly monoclonal after several weeks in culture, the same was true for U937_vec cells, and there was no evidence that the predominant vector integration event activated nearby genes in either of the two cell lines. It is therefore highly unlikely that cooperation with a gene activated by retroviral insertion facilitated the continuous proliferation, or any other phenotype, of U937_EVI1 cells. Furthermore, sequence analysis of the vector-borne *EVI1* cDNA in the nearly monoclonal state excluded the possibility that U937_EVI1 cells were able to proliferate due to a defect in the exogenous *EVI1* gene. Because *EVI1* has been shown to cooperate with *N-RAS* mutations in human and murine leukemia [60], [61], [62], [63], we sequenced the N-terminus of the *N-RAS* gene in U937_EVI1 cells, but found no alterations in the mutational hotspots in exons 2 and 3. At present, the possibility that a different mutation or a stochastic change in gene expression may cooperate with *EVI1* to allow normal proliferation of U937_EVI1 cells cannot be ruled out. In fact, the identification of such an alteration may help to explain how AMLs with overexpression of *EVI1* are able to maintain the increased proliferation rate associated with this disease, and will therefore be attempted in future studies. We are nevertheless confident that such a secondary alteration is not the true reason - rather than *EVI1* overexpression per se - for the most interesting phenotype of U937_EVI1 cells, namely their partial resistance to antileukemic drugs, because this phenotype could be reproduced in independently infected U937 cells (data not shown), as well as in HL-60 cells expressing *EVI1* in an inducible manner.

*EVI1* has previously been reported to inhibit apoptosis in response to several stimuli, including the anticancer agents taxol and IFN-α [23], [24], [25]. Nevertheless, until recently no data were available regarding a role of *EVI1* in resistance to drugs used in the treatment of AML. We now show that *EVI1* partially protected human myeloid cells from the cytotoxic effects of etoposide and the anthracycline daunorubicin, both of which induce DNA double strand breaks and are used in AML therapy. In fact, anthracyclines are one of the two pillars of current AML regimens and accordingly were also included in all of the large studies in which *EVI1* overexpression and/or 3q26 rearrangements were found to be associated with a poor prognosis [8], [9], [10], [11], [12], [60], [64], [65], [66], [67]. For some reason, however, we did not observe an increased resistance of U937_EVI1 cells to cytosine arabinoside (araC; data not shown), which is the mainstay of AML treatment. In contrast, Yamakawa *et al.* recently reported that knockdown of *EVI1* increased the sensitivity of UCSD/AML1 cells to araC, and proposed that the cell adhesion molecule integrin α6 (*ITGA6*) played a role in *EVI1* induced chemotherapy resistance: *ITGA6* was upregulated by *EVI1* and increased the adhesion of human hematopoietic cell lines to matrigel and stromal cells, and antibodies against ITGA6 decreased the viability of matrigel grown cell lines with high *EVI1* expression in the presence of araC [51]. Interestingly, however, experimental downregulation of *EVI1* enhanced cellular sensitivity towards araC also in suspension culture, a context in which a role of *ITGA6* would seem less obvious [51]. Therefore, and out of the general consideration that a single downstream molecule is unlikely to fully explain the phenotype caused by the expression of a transcription factor, additional *EVI1* targets are likely to contribute to the chemotherapy resistance induced by this oncogene.

Our microarray analyses revealed a substantial and highly significant overlap between gene expression changes elicited by *EVI1* expression and etoposide treatment, suggesting that *EVI1* overexpressing cells may be partially pre-adapted to the exposure to cytotoxic drugs. Among the genes that were upregulated by both *EVI1* and etoposide, *CDKN1A/p21/WAF* appeared of particular interest. *CDKN1A/p21/WAF* has been previously shown to be both upregulated by cytotoxic drugs and to protect from their effects, implying that its induction under these conditions represents a cellular defense mechanism rather than a pro-apoptotic event. This has been amply demonstrated also for hematopoietic cells [29], [30], [31], [33], [35], [36], [37], [39]. Pathological overexpression of p21 was associated with chemotherapy resistance and poor prognosis in several tumor types, including AML [26], [27], [28]. In addition, *CDKN1A/p21/WAF* has been reported to be induced in response to several leukemia associated oncogenes and to play a role in protection from DNA damage in this context [35], [40], [41]. According to a very recent report, *CDKN1A* was upregulated by *EVI1* in the human osteosarcoma cell line U2OS [57]. In the experimental system presented here, *CDKN1A/p21/WAF* was induced by etoposide and by *EVI1*. Interestingly, the p21 protein was located mostly in the cytoplasm both in the absence and presence of *EVI1*, and even in cells experimentally expressing this protein, agreeing with the fact that the antiapoptotic activity of p21 was associated with a cytoplasmatic location in many cases [26], [27], [34]. Experimental overexpression of p21 in U937_vec and U937_EVI1 cells mimicked the effects of *EVI1* overexpression in that it mediated partial protection from chemotherapeutic drugs.

Clearly, a full understanding of the molecular mechanisms through which *EVI1* confers chemotherapy resistance to AML and other malignant diseases will require further studies. The ultimate goal of these efforts is the development of new therapeutic options to improve the prognosis of patients with *EVI1* overexpressing malignancies.

## Supporting Information

Table S1 **Genes regulated by** ***EVI1*** **, etoposide, or both in U937 cells.**
(XLS)
